# A novel long term short interval physical activity regime improves body composition in mice

**DOI:** 10.1186/1756-0500-6-66

**Published:** 2013-02-19

**Authors:** Jorming Goh, Warren C Ladiges

**Affiliations:** 1 Interdisciplinary Program in Nutritional Sciences Medicine, University of Washington, Seattle, WA, USA; 2 Department of Comparative Medicine, University of Washington, Seattle, WA, USA

**Keywords:** Long term physical activity, Wheel running, FVB, C57BL/6, F1 hybrid mice, Fat mass, Lean body mass, Short interval running

## Abstract

**Background:**

Exercise training (ET) and physical activity (PA) offer obvious health benefits in regular participants. In pre-clinical animal models, treadmills and running wheels are the models of choice for intervention studies using ET and PA. However, the frequency and duration necessary for positive effects on health are not completely understood. We investigated the impact of short duration voluntary wheel running on body composition in FVB × C57BL/6 F1 hybrid mice over 22 weeks. Mice were randomized and given access to voluntary wheel running (N = 6) or locked wheels (N = 5) for 1 hour per night during the dark cycle, 5 days per week.

**Finding:**

Average weekly running distance was generally cyclic in nature over the 22 weeks but did not change significantly from week to week, except for a difference between week 3 and week 9 (P = 0.05). Daily running distances ranged from 0.78 km to 1.45 km. Compared with non-runners, runners demonstrated significantly lower relative fat mass (9.98 ± 0.56% vs. 14.91 ± 1.47%, P = 0.0067) and significantly higher relative lean mass (79.18 ± 0.65% vs. 75.41 ± 1.28%, P = 0.019). No differences were observed with respect to glucose metabolism.

**Conclusion:**

Voluntary wheel running for one hour a day five days a week over a five month period improved body composition in young adult mice. This repetitive short interval exercise regime should be a useful model to investigate the effects of structured moderate intensity physical activity on physiological performance and chronic disease conditions in mice.

## Introduction

Regular participation in physical activity or exercise is beneficial for health promotion and disease prevention. The definition of physical activity is “skeletal muscle contraction resulting in increased energy expenditure above basal levels”, whereas exercise is defined as “a subset of physical activity, where it is planned, repeated and structured, with the goal of enhancing or maintaining physical fitness [1].

Many investigators have explored the role of physical activity or exercise training on different chronic diseases using laboratory animals. Typical modes of physical activity or exercise training have been either the voluntary wheel running system or the motorized treadmill. Each has its own merits and disadvantages. The motorized treadmill allows investigators to manipulate the intensity (incline and speed) and duration of running. However, because the mice are being coerced to run, this model may not be an accurate representation of running behavior. The advantage of voluntary wheel running is that the intermittent start-stop nature is characteristic of the running behavior in mice and thus, a useful model to simulate their natural physical activity [2]. A disadvantage is the variability in the amount and intensity of the running performed by the mice on the wheels [3]. The ability to control the “dose” of physical activity in terms of duration, frequency and intensity is therefore lost.

The purpose of this study was to determine whether group housed mice given access to running wheels for the first hour of the dark cycle, when mice are normally active because they are nocturnal animals, 5 days a week would attain any physiological benefits, with respect to body composition and glucose metabolism, compared with non-running control mice.

## Methods

### Mouse model

The experimental protocol was reviewed and approved by the Institutional Animal Care and Use Committee (IACUC) at the University of Washington, Seattle. FVB males were bred with C57BL/6 females to produce F1 hybrids. Females were used in the running and non-running conditions and housed three to five per cage, except for a one hour running wheel session, 5 days a week, in a standard rodent room within a specific pathogen free (SPF) barrier facility with 12-hour dark and light cycles. Ambient temperature in the room was maintained at 21–25 degrees C. Mice were fed standard rodent chow (5053; Picolab, Richmond, IN) and water *ad libitum*.

### Wheel running design

At 90 days of age, mice were randomized into running and non-running groups. Mouse numbers of at least five per group were needed, based on 80% probability of detecting a 10% difference in body weight at P = 0.05, with a standard deviation of 1. Runners were removed from group housing at the beginning of the dark cycle (6 PM) and placed in individual housing with access to freely rotating running wheels. Non-runners were treated in the same manner, wherein they were placed in individual housing with access to locked running wheels. The first hour of the darkness was selected because we had observed these mice to be active at the very beginning of the dark cycle. Mice are nocturnal and naturally active at night. One-hour running activity was monitored continuously for the entire duration of the study using running wheels (diameter: 15.5 cm) that transmitted electronic signals wirelessly to a monitoring hub (*Med Associates*, ENV-044, Vermont). Time spent on running wheels as well as the number of revolutions was captured every minute. Running activity performed by each mouse was recorded for every session, and weekly body weights recorded. Both groups of mice were kept in this set-up for one hour, five days per week for a total of 22 weeks, at which time they were euthanized *via* carbon dioxide asphyxiation and necropsied to rule out any grossly observable lesions.

### Quantitative magnetic resonance (QMR) analysis of body composition

Body composition was determined one week before running was started and one week before the study terminated. QMR (*Echo Medical Systems, Houston, TX*) was used to quantify lean and fat mass *in vivo*. In brief, mice were placed while conscious, into a plastic sample holder. This sample holder was then inserted into the magnetic resonance machine. A total of 3 measurements were obtained for each mouse and used in subsequent statistical analyses.

### Glucose tolerance test

At the end of 5 months, mice were fasted overnight for 16 hours prior to the intraperitoneal glucose tolerance test (IPGTT). Mice were injected with 20% D-glucose (G7021; Sigma Aldrich) in phosphate buffered saline (14190; Invitrogen) at a dose of 2 g glucose/kg body weight. Blood glucose measurements were obtained *via* tail vein venipuncture at measured with a glucometer and Comfort Curve Test Strips (Advantage; Accu-Check, Roche, Basel, Switzerland) at 0, 30, 60, 90, 120 and 240 minutes after injection.

### Statistical analyses

Student’s t-test was used to detect significant differences between groups for the following dependent variables: body weights, lean mass and fat mass. Changes in body weights and running distance were assessed using a repeated measures analysis of variance (ANOVA) with Tukey’s post-test analysis. Prism (Graph Pad) was used to perform the statistical analyses. All data are presented as means ± standard deviation. Statistical significance was set at P ≤ 0.05.

## Findings and discussion

**Figure 1:**
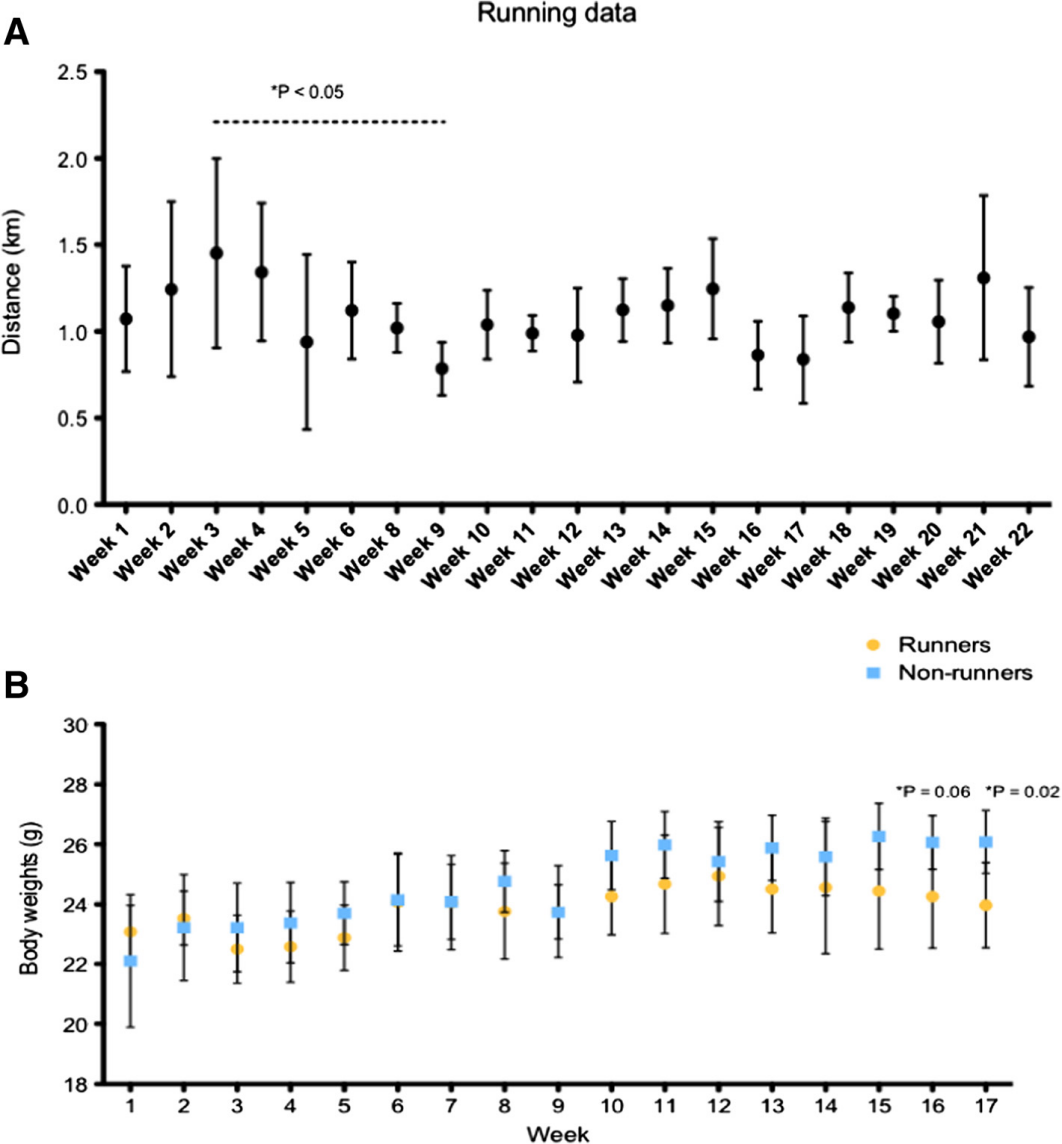
**The average weekly distance ran was generally similar during the course of the study, and body weights between runners and non-runners did not diverge until after 4 months. A.** There was no significant variation in mean weekly distance ran, except for the 3rd week, which was significantly greater compared with the 9th week. **B. **Body weights of both runners and non-runners were measured weekly during the 5 month running period. A trend but non-significant change in body weights between the two groups was first observed during the last week of the fourth month (P = 0.06), followed by a significant change the first week of the fifth month (P = 0.02).

The mean distance ran by each mouse per week was 1.085 ± 0.16 km, with a minimum distance of 0.78 km and a maximum of 1.45 km per day (Figure 1A). Mice in the running group attained this maximum distance of 1.45 km in week 3, and the minimum distance of 0.78 km in week 9. In the last week of the study (week 22), the mice ran a mean distance of 0.97 km. With the exception of weeks 3 and 9, the average distance ran was not significantly different between all other weeks. The cyclical nature of their running patterns suggests that motivation to run is an underlying factor. Alternatively, the mice could be slowing down from previous bouts of physical activity in the weeks where they ran longer distances.

**Figure 2:**
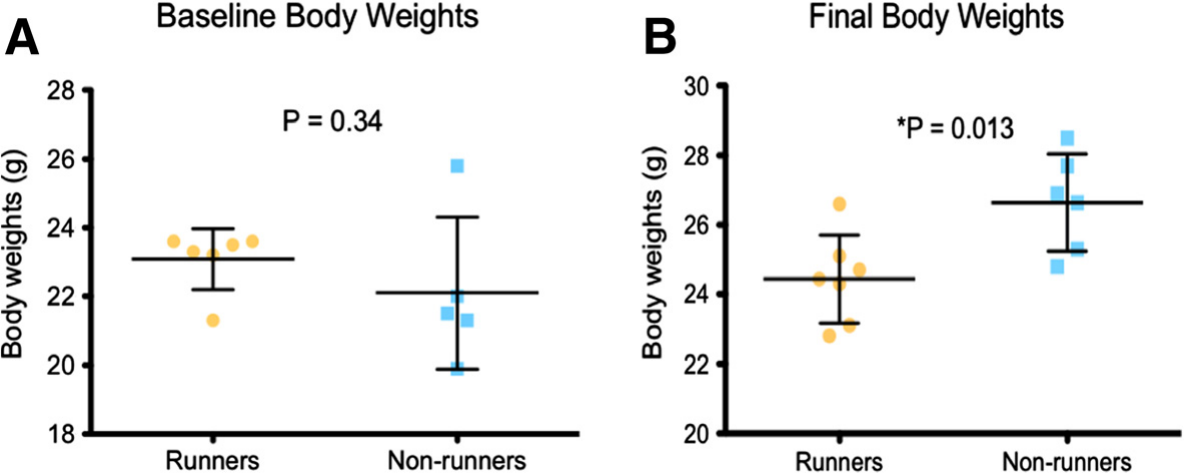
**Voluntary wheel running resulted in decreased body weight. A. **Baseline body weights of runners and non-runners were measured before the study began and no significant differences were observed (P = 0.34). **B. **Runners demonstrated decreased body weight at the end of the study, compared with non-runners (P = 0.013).

**Figure 3:**
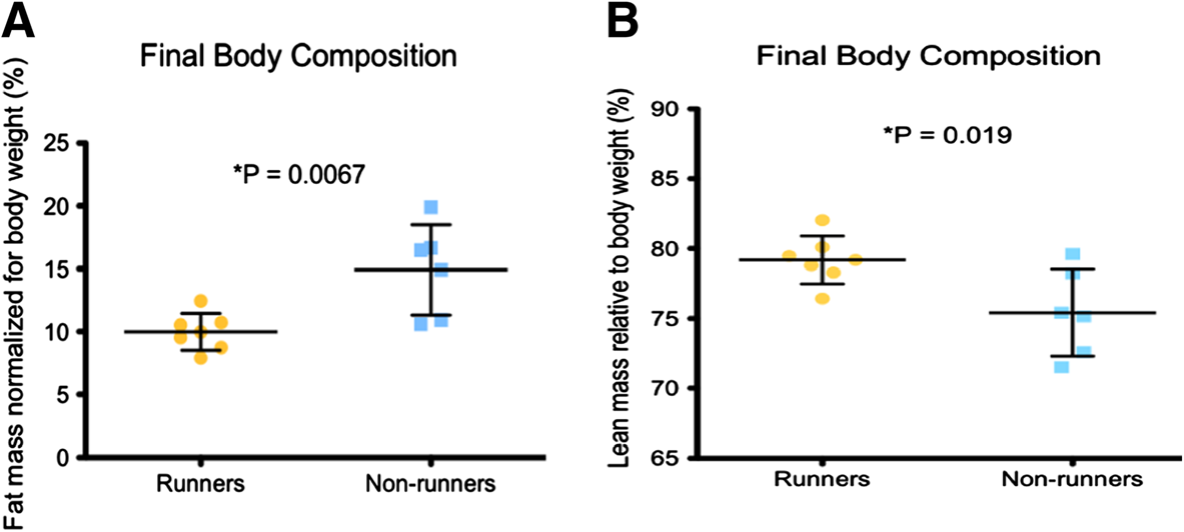
**A Regular physical activity reduced adiposity and enhanced lean body mass. A. **After 5 months of running, runners demonstrated reduced relative fat mass, compared with non-runners (P = 0.0067). **B. **Runners also exhibited increased lean body mass compared with non-runners (P = 0.019).

Significant changes in body weight between runners and non-runners were not seen until near the end of the study, which was after 17 weeks of running (Figure 1B). Runners and non-runners demonstrated similar body weights (23.08 ± 0.36 g, 22.10 ± 0.98 g, respectively) at the beginning of the study (Figure 2A). At the end of the study, the mean body weight of runners was 24.43 ± 0.48 g compared with 26.64 ± 0.57 g in non-runners (P = 0.013). Relative fat mass was decreased to 9.98 ± 0.56 per cent in runners compared to 14 ± 1.47 per cent in non-runners (P = 0.0067) (Figure 3A), while relative lean body mass was increased in runners to 79.18 ± 0.65 per cent compared to 75.41 ± 1.28 per cent in non-runners higher (P = 0.019) (Figure 3B). Our results suggest that changes in body composition may require up to 5 months of running, 5 days per week for an hour in this set-up. In a study designed to address the effects of exercise and age on serum cytokine and chemokine profiles [4], treadmill running for 4 weeks did not influence differences in body mass between young, exercise-trained C57BL/6 males or controls. We suggest that adding additional weeks of running in our study allowed a positive effect.

Despite a significant improvement in body composition, runners did not show differences in glucose tolerance compared with non-runners (data not shown). This is likely because both groups of mice were healthy young adults and should not be glucose intolerant at this stage of their lives. Alternatively, it is possible that the “dose” of one hour of wheel running may be insufficient to induce any differences in glucose metabolism between runners and non-runners that are young and healthy. In a similar study, 6 months of voluntary wheel running attenuated body weight gain and white adipose mass in active, female A/J mice and not in sedentary mice [5]. Further, in spite of the positive effect on body composition, there were no differences between either group in plasma glucose, insulin or total cholesterol concentrations. Mice were 6 weeks of age when the study began and were 7 months of age when it ended. This end point was similar to mice in our study, where they were 8 months of age when the study was terminated.

A disadvantage of voluntary wheel running for scientific studies is the inability to control the time and duration of running. We have overcome this disadvantage by demonstrating that 5 months of voluntary wheel running for 1 hour a day, 5 times per week, has the effect of reducing adipose mass and increasing lean body mass, thus contributing to improved body composition. This set-up would most likely simulate the US Surgeon General’s recommendation of 30 minutes of moderate-intensity physical activity for health promotion for people. Sedentary behavior, particularly sitting, is positively correlated with increased mortality from cardiovascular disease as well as from all other causes, in a dose-dependent manner [6]. Our data also show that the experimental set up is useful for testing group housed animals, where individual mice can be temporarily separated for the purpose of a controlled “dose” of physical activity. This provides a less stressful housing environment than when mice are continuously contained in single cage housing, since they are social animals by nature.

In conclusion, this study demonstrates that voluntary wheel running can be modified into a controlled and structured form of physical activity, and used as a pre-clinical model to improve body composition. The unique experimental set up should be useful for long-term physical activity studies in mice, particularly those that are designed to explore the role of exercise in chronic disease intervention and aging.

## Competing interests

The authors declare they have no financial or non-financial competing interests in relation to this manuscript.

## Authors’ contributions

JG conceived the design of the study, performed data collection, analyzed the data and drafted the manuscript. WCL conceived the design of the study and assisted with drafting the manuscript. Both authors read and approved the final version of the manuscript.
